# The Clinical Implications of Ashwagandha (*Withania somnifera* L.) with a Special Reference to Side Effects—A Review

**DOI:** 10.3390/nu18050871

**Published:** 2026-03-09

**Authors:** Kaj Winther

**Affiliations:** Department of Nutrition, Exercise and Sports, University of Copenhagen, 1958 Frederiksberg C, Denmark; kaha@nexs.ku.dk; Tel.: +45-31333067

**Keywords:** ashwagandha, nutrients, herbal supplement, efficacy, side effects

## Abstract

Ashwagandha (*Withania somnifera* L.) root powder and extracts have long been used in Ayurvedic medicine to improve sleep and anxiety. Recent scientific investigations into its efficacy have shown promise for relief from anxiety, insomnia and stress and for improving the immune system. It has also been suggested that oxygen uptake in the cardiovascular system, muscle strength, cognitive function, the reproductive system and the aging process significantly benefit from ashwagandha treatment. Since the herbal remedy is taken daily by millions of people in India, China and parts of the West, it is interesting that there are very few case reports of side effects directly attributed to the treatment, suggesting that the administration of ashwagandha preparations may be safe. Currently, neither the European Medicines Agency nor the FDA considers ashwagandha as a drug or general health supplement. Therefore, ashwagandha products are marketed in the West as dietary supplements so that users may be exposed to unscrupulous vendors. In this narrative/literature review, scientific findings from basic research and human clinical trials on herbal remedies, spanning the period from 1994 to date, were critically evaluated for the purpose of highlighting knowledge gaps to provide context for new research. Such investigations will provide evidence for the efficacy and safety of ashwagandha treatment, thus making the herbal preparations more accessible to a wider audience.

## 1. Introduction

Ashwagandha (botanical identification: *Whitania somnifera* (L.)), also known as Dunal or winter cherry (in English), is of the family Solanaceae, like potato and tomato. The leaves and stems from members of the Solanaceae family, which also include auberges, can be harmful and even toxic [1] due to high concentrations of glycoalkaloids and should not be eaten. However, the potato and the fruits from tomato and aubergine plants are known and used worldwide for their nutritional advantages. Ashwagandha has been known in the Ayurvedic tradition for almost 4000 years, with the leaves and stems being avoided due to their high content of cytotoxic and liver-damaging phytochemicals. By contrast, ashwagandha root, in the form of a powder or as a water extract, is very useful as a food and herbal remedy and does not have side effects [2].

Ashwagandha is often referred to as “Indian ginseng”, though this is very misleading, as the plant has genetically nothing in common with ginseng. Ashwagandha is a large family consisting of many different morpho- and chemo-subspecies [3]. So, when speaking about ashwagandha, one should be aware of the huge variety in appearance and in the phytochemical content of the ashwagandha subspecies [3,4].

**Figure 1:**
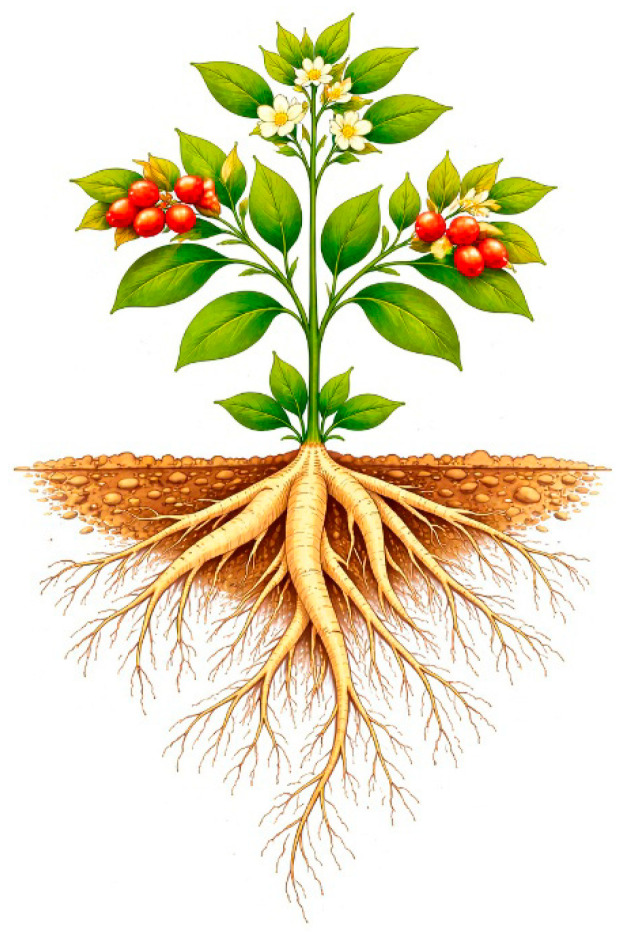
Ashwagandha plant, including the root. As is the case for other plants of the Solanacea family, the roots of the ashwagandha plant are not poisonous.

Ashwagandha is a perennial plant that grows well in India and in most Asian and African countries, as well as in Europe, mostly in Spain. The plant is an erect subshrub with a green stem and leaves and yellow or greenish flowers. It flowers all year, and the flowers are followed by small orange-reddish berries containing seeds (see Figure 1). Ashwagandha normally grows about 75 cm high but can be up to two meters high. It grows mainly on wasteland up to an altitude of 1800 m, but can also be cultivated in the lowlands, where the root is used as a nutritional supplement [2,3,4,5,6].

The fresh roots are pale to yellowish-brown with a creamy interior. A main root often bears much smaller secondary roots, which are normally removed before the main root is dried and powdered (Figure 1). The resulting powder is light grey to yellowish-brown. Commercial ashwagandha can be sourced from wild or cultivated plants. There are at least five different cultivated species in India, and other species of ashwagandha are also cultivated in the United States of America [2,3,4].

### 1.1. The Evolution and History of Ashwagandha

The species name “*Somnifera*” refers to the fact that in old times, the plant was used to promote sleep. Later in history, the plant was also known for its ability to generate energy and modify stress. The root has a strong aroma that may direct our thoughts towards horses, for “Ashwa” literally means horse in Sanskrit, while “ghanda”, in the same language, means smell. This can project the idea that the plant will make you “strong like a horse”. As the name suggests, ashwagandha has been used in ancient Ayurvedic traditional medicine for centuries for treating different ailments, including tiredness, sleep disturbances, lack of energy, stress, hormonal disruptions and lack of sexual drive.

### 1.2. Utilization in Modern Times

During the last 50 years, interest in ashwagandha and especially the root of the plant has drawn increasing attention from scientists. Applications of modern technology in examining the constituents and effects of the plant parts have shown that extracts, especially of the root, can improve sleep, as well as mediate analgesic, antiarthritic, anti-inflammatory and muscle-strengthening effects [3,4,5,6,7]. Results from these studies and the accompanying clinical studies, which also show promising results on stress, including a lowering of cortisol and a normalization of serum TSH and thyroid hormones in hypothyroid patients [8], are reviewed below in Section 3.

## 2. Content of Different Metabolites in Ashwagandha Root and Leaves

As stated earlier, only the main tap root, which is cylindrical and about 1 to 1.25 cm in diameter, is traditionally used for medicinal purposes. Smaller and more peripheral root fragments are discarded as they contain less of the active ingredients [2,3,4]. Traditionally, the leaves were only used in topical applications or for treating specific conditions under the supervision of Ayurvedic doctors.

An example of the wide variety of metabolites detectible in ashwagandha roots, leaves and fruits is presented in Table 1 [9,10,11,12,13]. There is a distinct quantitative difference in the contents of secondary metabolites extractable from the leaf, as compared to those from root parts. For example, withanone (class—steroidal lactones; a subclass of triterpenoids) is about six times higher in the leaves than in the root, while withaferin A (a more toxic withanolide) is more than twenty times higher in the leaves than in the root (Table 1). Unfortunately, withanolides are often declared as one group on the labels of commercial ashwagandha preparations, whereas, as shown in Table 1, there are several types of steroidal lactones, each with differing biological effects. This practice makes it impossible to determine if leaves, which are rich in withaferin A and withanone, have been included in the so-called ashwagandha root product.

As shown in Table 2, the type of solvent used in the preparation of the plant extracts is also very important [9]. This means that it matters if an extract was made with alcohol (e.g., methanol) or water or a combination of both, since there are water-soluble, as well as fat-soluble, phytochemicals from the same ashwagandha plant. The same paper [9] also analyzed the phytochemical classes and went on to investigate the primary and secondary metabolites. For example, they identified porphyrin metabolites like chlorophylls and their degraded secondary metabolites, pheophytins (not included in the tables). These secondary metabolites are formed during the extraction process, where chlorophylls and pheophytins are observed in leaves and not in roots.

Although most products containing ashwagandha are declared as root-only products, it is not uncommon to find unscrupulous vendors partially replacing or augmenting the expensive root material with the significantly cheaper and easier-to-obtain aerial parts. As previously stated, ashwagandha leaves are rich in ingredients like withaferin A and whitanone, which can exert liver injury. Therefore, extracts from leaves should never be a part of an ashwagandha product.

### 2.1. The Need for Standardization for Ashwagandha Root Preparations

The main active ingredients in ashwagandha are the steroidal lactones or withanolides, and not surprisingly, six different types are the basis for the standardization of some leading products, like KSM66, which is by far the most sold and researched product worldwide. However, some other phytochemicals that may be contributing to the activity can be polyphenolic compounds and flavonoids. So, when speaking about standardization, the above-mentioned ingredients are candidates that should be used. In addition, the toxic withanone and withaferin A, which occur in high concentration in leaves, may be used for standardization. Ideally, these ingredients should be at very low concentrations in prospective ashwagandha products. Better still, they should be below detection levels to ensure that ashwagandha leaves or other unsafe ingredients are not included in the formulation under consideration.

### 2.2. The Importance of Standardization

Root content of alkaloids: Data from 15 very promising genotypes of ashwagandha showed that the total root content of alkaloids differed from 0.24% to 0.90% dry weight [14]. This study was followed by a similar study [15], where 25 different genotypes of ashwagandha root showed a variation in alkaloids from 0.16 to 0.96% (dry weight), indicating that when generating herbal medicine, the species chosen is also of great importance. This pattern with alkaloids was also observed when the total amount of withanolides was investigated. Here, the variance was from 0.23 to 1.11% when comparing levels in different cultivated ashwagandha and some wild varieties [16]. The amount of starch and fibers also showed a wide variation when 25 different genotypes were tested [15]. These data clearly indicate the importance of the subspecies chosen, in addition to the extraction methodology used when making the preparation.

As with all plant-sourced materials, the quality of the ashwagandha product is dependent on the soil properties, the use of fertilizers or manure, the amount of rain that falls during the growth process, the number of sunny hours, the altitude where the plants grew and the extent to which the plant matured before being harvested. For all these reasons, when determining what ashwagandha product one should choose, key factors to be considered, especially as regards the criteria used for the standardization of the product, should include natural variability in plant composition (due to genetics), environment and processing.

### 2.3. Are Raw Materials and Formulations of Commercial Ashwagandha Preparations the Same?

It is often puzzling when one goes to a store or a marketplace to buy a potato or an apple, where there are so many different species that taste different and perhaps do not contain the same ingredients. For just as with grapes used for making wine, the quality of the vegetables can differ from year to year and from location to location.

Similarly, when one buys an ashwagandha root product, one ought to be watchful of the subspecies of the source plant, where it was grown, when it was harvested and how the finished herbal remedy was prepared because of the factors outlined above in Section 2.2. There are many wild ashwagandha species around the world. In India, where the plant is cultivated, there are at least five different species grown, and the same is true for the USA [2,3,4]. The tables presented in this review include the results of analyzing metabolites of subspecies that have been cultivated for two decades by an Indian family-owned company and marketed under the name “KSM66”. The KSM66 is extracted using a very standardized extraction procedure, involving the use of milk and water. Other established products in the market may have used ethanol, methanol or combined extractions, resulting in totally different results, as shown in Table 2. So, when considering whether a particular ashwagandha preparation is relevant or not, or when discussing if ashwagandha should be avoided due to possible side effects or not, two pertinent questions to be considered are as follows:

1. Is the product a good-quality ashwagandha root extract?
2. Are there chemically testable assurances that the product does not contain contaminations by elements from other plant parts or materials that are not from the ashwagandha root?

Given the wide range of dietary supplements and the varying quality of products in the market, it is preferable to choose a clinically well-documented and characterized product.

## 3. The Impact of Ashwagandha Root Preparations on Health and Disease

### 3.1. Ashwagandha’s Influence on the Immune System

The immunomodulatory effects of ashwagandha, defined as an increase in IgA, IgM, IgG, IgG2, IgG3 and IgG4, were seen in a placebo-controlled study in which humans were administered the herbal medicine for 30 days [17]. Aside from the effect on immunoglobulin content, there was also a significant improvement in cytokines like IFN-gamma and IL4 and in CD45+, CD3+, CD4+, CD8+ and CD19+, as well as in natural killer (NK) cells. No such changes were observed in the placebo group. It is important to note that all cellular and biochemical markers of the immune system, which were improved, were still within the normal physiological range of the respective markers, indicating that the treatment did not give rise to uncontrolled immune stimulation. In a study with human monocyte cells, ashwagandha inhibited TNF alpha, IL-1 and superoxide production in a dose-dependent manner [18]. Such findings were reinforced in an animal study with rats, where a dose-dependent reduction in CRP and oxidative stress was evidenced upon ashwagandha administration, as indicated by a decrease in lipid peroxidation and in glutathione-S-transferase activity [19]. In another in vivo rat model, where gout was induced by monosodium urate crystals, edema was reduced to normal levels, indicating a potent anti-inflammatory effect with ashwagandha treatment. In contrast to the control group treated with NSAID, there were no gastrointestinal side effects, indicating that ashwagandha can retard amplification and propagation of the inflammatory response without causing any gastric injury [20].

### 3.2. Ashwagandha’s Influence on the Cardio-Respiratory System, Oxygen Uptake, Muscle Strength and Recovery

In a rat model with isoprenaline (isoproterenol)-induced myocardial necrosis, *Withania somnifera* exerted cardio-protective effects [21]. An augmentation of endogenous antioxidants, maintenance of the myocardial antioxidant status and significant restoration of most of the altered hemodynamic parameters, initiated earlier by the artificially induced myonecrosis, were suggested as sources of the observed cardio protection observed with the treatment. In addition, there was improved cardiorespiratory endurance in healthy younger athletes in a placebo-controlled study of ashwagandha water extract (KSM66) administered for three months [22]. The study, which assessed the maximal oxygen uptake and self-reported quality of life, showed a great and statistically significant improvement in VO2 max (maximal oxygen uptake/consumption) after a 20 m shuttle run test and in quality of life (Qol), which was associated with KSM66 treatment. These data were also supported in a double-blinded, randomized, placebo-controlled study with 50 athletes, in which VO2 max, recovery, energy and the ability to manage stress were improved as a result of ashwagandha treatment (KSM66) [23]. The same was observed in an earlier study (2010), in which a group of young adults in a placebo-controlled clinical trial used ashwagandha for 8 weeks [24]. In a double-blinded study, where younger adults were treated with ashwagandha for two months, muscle strength and recovery after strenuous work were tested. In accordance with the above-reported studies, the ashwagandha (KSM66)-treated group had a highly significant improvement in muscle strength when tested with the bench press exercise and leg extension exercise. Furthermore, the volunteers also developed a significantly greater muscle size and improved their endurance and VO2 max [25]. Compared to placebo, ashwagandha-treated healthy volunteers showed a significant decline in exercise-induced muscle damage as indicated by serum creatinine kinase measurements, combined with an increase in serum testosterone and a decline in body fat percentage [26]. Muscle force and serum contents of total cholesterol were likewise improved when healthy volunteers were treated with up to 1250 mg of ashwagandha in one month [27]. An improvement in muscle recovery, as well as in levels of free and total testosterone, was also documented with daily administration of 600 mg daily to healthy younger volunteers for two months [28]. The above studies are summarized by a meta-analysis on physical performance and ashwagandha, concluding that ashwagandha supplementation was more efficacious than the placebo for improving variables related to physical performance in healthy men and females [29]. Recently, a 12-month study comprising 191 healthy volunteers with an age range of 18–65 years old, treated with 600 mg of ashwagandha KSM66 daily, likewise showed an improvement in serum testosterone levels, possibly due to a stress-reducing effect. It is important to state that although the levels are modified, they remained within normal range [30].

### 3.3. The Impact of Ashwagandha on Stress, Anxiety, Insomnia and Sleep

Ashwagandha (full-spectrum) root extract was tested in volunteers under chronic stress in a randomized, double-blind, placebo-controlled set-up. The study included measurement of stress using standard stress and anxiety questionnaires. Serum cortisol was measured throughout the study, which lasted for 8 weeks. The ashwagandha root extract group showed a highly statistically significant reduction in stress after 8 weeks of treatment when compared to the placebo. In accordance with the stress reduction, serum cortisol dramatically declined [31]. It should also be noted that there was a significant reduction in anxiety and insomnia because of the treatment, in addition to a decrease in social dysfunction and depression, as revealed with participants’ responses in GHQ-28 questionnaires. A normalization of serum cortisol and significant decline in stress levels (when evaluated through questionnaires) were also documented in another placebo-controlled, randomized, double-blind study [32]. In a similar study, using the Hamilton anxiety rating scale, ashwagandha root extract treatment also resulted in a statistically significant lowering of anxiety and in a normalization of serum cortisol [33]. A statistically significant reduction in serum levels of cortisol, a reduction in the level of stress and improvement of sleep were also observed when 60 and 50 middle-aged volunteers, represented by both sexes, were tested for 8 and 12 weeks, respectively [33,34,35,36,37]. An alleviation of stress, anxiety and improvement of quality of life, as well as a (lowering) normalization of serum cortisol, in addition to an increase in blood levels of serotonin, was reported [38]. The above summary of the impact of ashwagandha on stress, anxiety and sleep is further supported by two systematic reviews and meta-analyses of randomized controlled trials [39,40]. As an anecdote, it should finally be mentioned that even in randomized, placebo-controlled studies in cats and dogs, ashwagandha treatment for one month resulted in a (lowering) normalization of serum cortisol and in a decline in fear and anxiety [41,42].

### 3.4. Ashwagandha and Cognitive Function

The efficacy and safety of ashwagandha root extract on affecting cognitive function in healthy stressed adults was tested in a randomized, double-blinded and placebo-controlled clinical trial, involving 130 men and women. The results showed that 300 mg of the root extract taken daily significantly improved memory and focus, psychological well-being and sleep quality, as well as reduced the level of stress. Aside from these positive effects on general well-being, the treatment, which lasted for three months, was safe, without reports of side effects [43]. In another efficacy and safety study, ashwagandha root extraction was tested in volunteers with moderately impaired cognition [44]. After 8 weeks of study, the ashwagandha-treated group (300 mg daily) showed statistically significant improvements in immediate and general memory. The treated group also showed enhancements in executive function, sustained attention and information processing speed. Ashwagandha extraction was later tested in Wistar rats of both sexes. Treatment resulted in increased brain function, as shown from electrophysiological changes in the delta and gamma bands, and the changes were statistically significant [45,46]. It is generally agreed that herbal remedies need to be used for some time before any impact of relevance can be measured, as herbal remedies generally do not work within hours. Very interestingly, it turned out that acute supplementation with ashwagandha (400 mg) improved selected measures of executive function, helped to sustain attention and increased short-term memory in test subjects [47]. There were also indications that administration of 225 mg of ashwagandha (nearly half the test dose) can improve measures of memory, attention and executive function while decreasing the perception of tension and fatigue in younger healthy individuals [48].

### 3.5. Ashwagandha and the Reproductive System

#### 3.5.1. Ashwagandha in Women

Ashwagandha has been credited with improving women’s sexual function. In a double-blinded, randomized, placebo-controlled clinical pilot study, 50 women were either treated with ashwagandha extract (600 mg daily) or a placebo for eight weeks. Sexual function was evaluated using psychometric scales such as the Female Sexual Function Index (FSFI) and Female Sexual Distress Scale (FSDS). When compared to the placebo, the ashwagandha extract led to improvements in the registered arousal, lubrication, orgasm and satisfaction [49]. This study was later confirmed in 80 women who were treated with a similar dosage and for the same period. Assessments were made using the same questionnaires [50]. The mechanisms behind the significant changes observed in the blinded studies and evaluated using questionnaires gained some biochemical support, as a normalization of estrogen levels was observed in peri-menopausal women [51]. This contrasts with a study on younger women still having their menstrual period, in which ashwagandha did not cause any changes in estrogen levels [52]. However, the normalization of symptoms reported for peri-menopausal women can have many possible reasons. Ashwagandha has been reported to improve sleep, lower stress and impact several brain functions detectible from electrophysiological changes in electroencephalograms [45,46]. This may impact sexual function. Moreover, ashwagandha contains isoflavones and flavonoids that may be estrogen analogs [53]. Ashwagandha extract was also shown to exert GABA mimetic properties and increase the secretion of gonadotropin hormones [54].

Indeed, ashwagandha treatment alleviated menopausal symptoms such as hot flushes, somato-vegetative and urological domain problems in peri-menopausal women. In the same study, there was a significant increase in serum estradiol and no significant changes in serum testosterone when compared with the placebo [51].

Traditionally, ashwagandha root has been used to prevent miscarriages and to stabilize the fetus. Despite this, during the last 20 years, there has been a growing suspicion that ashwagandha can cause miscarriage—the primary reference has most often been the World Health Organization (WHO), which, in turn, cited the American Herbal Pharmacopoeia (AHP) Ashwagandha Root Monograph and Therapeutic Compendium [2]. However, according to the American Herbal Pharmacopeia, the WHO monograph is an example of what is known in the medical literature as “citation distortion” since it did not fully articulate the AHP review, which stated the following: “There are conflicting reports regarding the use of ashwagandha in pregnancy. Large but undefined doses have been reported to possess abortifacient activity” [55]. The statement can be considered controversial since ashwagandha is traditionally used in Ayurvedic practice to prevent miscarriage and stabilize the fetus [56,57,58]. The latter notion is further supported by a recently published animal study, where pregnant Wistar rats received up to 2000 mg of ashwagandha extract per kg of body weight per day during the gestation period without showing any negative impacts on the reproduction process or on fetal development [59]. Finally, the Ministry of Ayush (Government of India) released a safety dossier in 2024 [60], noting the lack of abortifacient activity in ashwagandha root, citing all clinical and preclinical data that have investigated the use of ashwagandha and its root preparations in pregnancy. The conclusion was that no clinical or preclinical investigations revealed an abortifacient activity of ashwagandha root extracts, a claim which is supported by the American Herbal Pharmacopoeia (AHP) press release, 25 June 2024 [56].

#### 3.5.2. Ashwagandha in Men

From 2009 to 2024, there are several published scientific articles claiming that sexual activity, sperm count and motility, serum LH, FSH, testosterone levels and even erection and sexual arousal increased in sluggish male rats given ashwagandha for up to three months [61].

In conformity with animal studies, administration of 600 mg of ashwagandha extract daily for 2 months was also reported to elevate serum testosterone in healthy young male human volunteers [26], as well as in middle-aged overweight men [62]. Similarly, serum testosterone, sperm count and motility were significantly improved as a result of ashwagandha treatment—675 mg extract taken daily for 3 months—in a group of men under infertility screening, as well as in another group of men who were infertile [63]. Serum testosterone also significantly increased, as did sexual function, in a study on male volunteers with lowered sexual desire [64], who took 5 g of ashwagandha root powder for 3 months. In three other studies on infertile men, the quality of sperm improved, and the harmonic balance of seminal plasma metabolites and reproductive hormones increased [65,66,67]. By contrast, neither ashwagandha nor placebos provided any improvements when tested in volunteers with psychogenic erectile dysfunction [68]. Five studies performed in male volunteers with low sexual desire or infertility indicate an improvement in sex hormones, including serum testosterone, as a result of ashwagandha treatment. There are, however, papers on healthy younger men [26], and one on middle-aged overweight men [69], which, irrespective of the age, show a modest but significant increase in serum testosterone levels as a result of 2–3 months of ashwagandha treatment. Interestingly, the initial level of testosterone was within normal range when the study started and remained so throughout the duration of treatment. As will be discussed below in Section 3.8, this increase in testosterone with ashwagandha treatment is a slightly different pattern than what was observed for thyroid hormones, in which individuals with hypothyroidism before ashwagandha treatment were normalized, while subjects starting with normal thyroid hormone levels remained unchanged at the end of the experiment [8,41].

Therefore, the mechanism or biochemical basis for the modest rise (up to 15% increase in serum testosterone) in the above studies [26,69] and in another study, where the males were treated with ashwagandha, 600 mg daily for one year [29], is not clear. It should, however, be noted that increased physical activity can improve serum testosterone levels [70]. Thus, considering that ashwagandha treatment improves energy and physical activity in the recipient, the improvement of serum testosterone might indirectly result from volunteers becoming more physically active [26,69]. Furthermore, ashwagandha lowers cortisol levels in stressed subjects, which can, in turn, improve their testosterone levels [31].

### 3.6. Ashwagandha and Liver Protection

Like hepatoprotective green tea, garlic and red grapes, the root extract of *Withania somnifera* has been shown to improve superoxide dismutase and catalase levels, as well as enhance antioxidation in liver tissue [71]. In addition, ashwagandha was also shown to protect mouse livers from paracetamol-induced damage [72] and protect rats from gentamicin- and thioacetamide-induced liver damage (fibrosis and cirrhosis), as indicated by a significant reduction in the liver enzymes ALT and AST and a decline in bilirubin [73,74]. In the latter experiment, *Withania somnifera* administration exerted liver protection in rats by influencing the levels of lipid peroxidation products. Histopathological alterations after carbendazim-induced liver and kidney injury were also amended by 48 days of ashwagandha treatment in rats [75]. The root extracts of the herb were also shown to attenuate hepatic and cognitive defects in a rat model of hepatic encephalopathy induced by thioacetamide treatment [76]. Specifically, the administration of withaferin A (WA, a major constituent of ashwagandha roots; refer to Table 1) significantly improved the pathology of non-alcoholic steatohepatitis (NASH) in mice [77]. Here, WA prevented and therapeutically improved liver injury in two NASH mouse models, as revealed by lower serum aminotransaminases, hepatic steatosis, liver inflammation and fibrosis.

In old dogs, 28 days of ashwagandha treatment significantly improved ALT, AST and superoxide dismutase (SOD) and normalized glutathione (GSH) levels [78]. These changes in GSH levels can indicate how ashwagandha exerts its hepatoprotection, as it is one of the most important enzymes protecting the liver from elements that can be poisonous or destructive [79,80,81]. For example, oral administration of 300 mg of glutathione daily for 4 months to non-alcoholic fatty liver disease patients demonstrated a potential therapeutic effect of GSH, as indicated by a decline in the liver enzyme ALT [82]. In another study with healthy elderly dogs, ashwagandha root extract was also shown to significantly lower AST and ALT, in addition to supporting kidney function by improving creatinine and blood urea [83]. In a two-month study on healthy humans treated with 600 mg of ashwagandha (KSM66) daily, there were no changes in ALT and AST levels, indicating that the herb is neither hepatotoxic nor nephrotoxic under the tested conditions [26]. Similarly, in a long-term study that lasted 12 months, where 191 participants took 600 mg of ashwagandha (KSM66) daily, there were no clinically relevant changes in AST and ALT values [30].

However, there are a few case reports of liver damage, mostly from undefined ashwagandha extracts and often during co-administration with other unknown herbal remedies. This anomaly will be discussed below in Section 3.10, together with results from a recently published 12-month study, which focused on the side effects of ashwagandha treatment.

### 3.7. Other Ashwagandha Effects, Including Possible Impacts on Longevity

There have been some indications that ashwagandha may have antidepressant effects [84], although the mechanisms are not fully understood. Various compounds isolated from the root, stem and leaves have also been claimed to exhibit anti-cancer properties [3]. In randomized, double-blind, placebo-controlled studies, which involved testing a topical preparation of ashwagandha root, it was possible to demonstrate improved hair health [85] and enhanced facial skin in photoaged healthy adults [86]. These aging-related changes in skin have been reported to affect the anti-oxidative defense systems, like superoxide dismutase (SOD), catalase, glutathione and malondialdehyde in humans, as well as in animal models [87]. In the study on elderly dogs, it is interesting to note that in addition to improvements in liver enzyme (ALT and AST) levels, anti-inflammatory markers and indicators of the anti-oxidative defense system, such as SOD, catalase, glutathione and malondialdehyde, also improved with ashwagandha root extract treatment [83]. Some of these findings suggest a lifespan extension, which was also seen in another study where *Caenorhabditis elegans* were treated with ashwagandha root extract [88].

### 3.8. Ashwagandha and Thyroid Function, Including Thyrotoxicosis

Herbs like *Commiphora mukul*, *Humulus lupulus*, *Bauhinia purpura* and *Withania somnifera* (ashwagandha) are thought to interfere with thyroid function when consumed in their raw form or as extracts [89]. In a randomized, double-blind, placebo-controlled study, administration of 600 mg of KSM66 ashwagandha daily for eight weeks was found to normalize thyroid hormones in elderly volunteers with mild hypothyroidism [8]. The positive effect was accompanied by an improvement in the levels of T3 and T4 hormones, as well as a reduction in TSH [8]. This observation is particularly interesting because many of the elderly with cognitive impairment and a tendency for chronic depression often have a modest thyroid hypofunction as the underlying cause of their symptoms [90].

Since ashwagandha can improve thyroid levels in hypothyroidism, a very pertinent question is whether the treatment will continue to raise the hormone levels in volunteers with normal thyroid function. The result of such an undesired effect would be hyperthyroidism or an overactive thyroid gland, which is not desirable, as symptoms include tachycardia, nervousness or irritability, hand tremors, muscle weakness and excessive sweating. The frequency of hyperthyroidism in the general population, depending on where we are on our planet, is 1–2% [91]. As mentioned earlier in this review, many people in Asia, Europe and the USA are taking ashwagandha daily and have been doing this for centuries, with only very rare reports of hyperthyroidism. Hence, it is doubtful whether the few incidences of hyperthyroidism are due to ashwagandha alone or whether they have been provoked by diet or other factors.

As the possibility that ashwagandha might induce hyperfunction of the thyroid gland in some volunteers cannot be excluded, the safety of ashwagandha root extract (KSM66) (600 mg administered daily for 8 weeks) was tested in a randomized, placebo-controlled trial in 80 healthy volunteers, representing both sexes [92]. There were no indications that ashwagandha induced changes in T3, T4 or TSH in the actively treated group. Nevertheless, the study was only run for 8 weeks, and higher doses of ashwagandha could have been used. In 2022, another safety study was carried out, in which 2000 mg of KGM66 was administered daily to volunteers with normal thyroid hormone levels for 3 months. Results showed that the high doses of ashwagandha taken for three months did not lead to thyroid values outside the normal range or changes in TSH, T3 and T4 hormones [93]. In accordance with what has been reported so far in humans [8,93], results from experiments with several animal models support the notion that ashwagandha treatment improves thyroid function where hypothyroidism is induced experimentally but has no effect in normo-thyroid animals [94,95,96,97].

However, a literature search showed four rare cases claiming hyperthyroid function as a result of ashwagandha treatment. One case is a 47-year-old man from Japan, who two months after he started taking ashwagandha (dose not provided), suffered from minor weight loss (4 kg) and fatigue. There was no thyroid enlargement, but TSH was low, and T3 and T4 were raised [98]. Fifteen days after withdrawal of ashwagandha, symptoms and thyroid parameters that had been blamed on stimulation of the thyroid gland were normalized. In another instance, a 62-year-old female from the USA, who suffered stress, was diagnosed with thyrotoxicosis after self-administration of ashwagandha (1950 mg daily for two months). In addition to the ashwagandha treatment, the lady was also receiving estradiol (menopause) and self-administering collagen, astaxanthin, vitamin B complex and a “green powder”. She experienced physical fatigue, weight loss and palpitations two months after adding ashwagandha to the plethora of medicines she was taking. Her TSH was low, and T3 and T4 were raised. After discontinuation (time not given) of ashwagandha, the negative symptoms stopped, and thyroid parameters normalized. The suggested mechanism by the authors for the anomaly was direct stimulation of the thyroid tissue [99]. The third report involved a 73-year-old woman from the USA with hypothyroidism. Two years before presenting with the symptoms, she had stopped her prescribed levothyroxine treatment. Instead, she was self-medicating with ashwagandha treatment (dose unknown). At the time of the report, she came to the hospital with symptoms like palpitations, tachycardia, fatigue and hair thinning. Her TSH level was markedly reduced, but T3 and T4 were within normal range. Two weeks after withdrawal of ashwagandha treatment, the hypothyroid symptoms vanished, and the TSH level normalized. Nevertheless, three weeks following ashwagandha stoppage, the TSH level was elevated again, while T3 and T4 became low due to her baseline hypothyroid status [100]. The final case is a 32-year-old woman from Holland, who started out with ashwagandha (Holisan, Lelystad, The Netherlands; Lelystad capsules) at 250 mg daily for six weeks, followed by an increase to 500 mg daily. After a short while, she experienced weight loss and tachycardia. Upon admission into the hospital, thyroid hormone levels indicated thyroid dysfunction. Four weeks after discontinuation of ashwagandha treatment, hyperthyroid symptoms and thyroid values were normalized [101].

In conclusion, adverse side effects on the thyroid glands from ashwagandha treatment are rare. To the best of my knowledge, only four cases have been recorded. Although it is generally agreed that ashwagandha treatment improves thyroid function in volunteers with low thyroid function, while not affecting normo-thyroid patients, more detailed safety studies need to be carried out for clarification of this very important aspect.

### 3.9. Side Effects in General

It is well known that herbal remedies used in the Ayurvedic tradition can cause side effects, including liver toxicity [102]. Suspected hepatotoxic herbal remedies include the root of ginseng kianpi, a green tea, which can cause oxidative stress and apoptosis in liver cells; several lichen species; and the root of kava; and *Withania somnifera* (ashwagandha) [102,103,104]. However, as will be discussed below, the basis for the claim that ashwagandha can be hepatotoxic is conflicting [102,103].

In the clinical trials mentioned above in Section 3.6, where a total of 191 volunteers (male and female) took ashwagandha (KSM66) for up to 12 months, there were no indications that active treatment was different from the placebo, regarding side effects like itching skin, gastrointestinal complaints, drowsiness and headache [30]. In the same studies, there were no side effects related to the liver, as seen in changes in biochemical parameters suggestive of liver damage jaundice or pruritus, or to the thyroid gland, as seen in changes in biochemical markers, weight loss, tachycardia and hair loss. This trend is furthermore supported by all the review papers and meta-analyses cited in this review. It should, however, be noted that most of the clinical studies, on average, lasted from 1 to 3 months, which is not comparable to taking the remedy for years. In some of the cases, the side effects occurred after 3 months of the treatment. Indeed, in a single case, the negative effects were reported after more than one and a half years of treatment.

When the reported cases of side effects were evaluated by Professor Nilsson, a specialist in medicine and gastroenterology at the University of Lund in Sweden, he could not find a clear relation between ashwagandha preparations and liver ailment [105]. His conclusion was that ashwagandha, in rare cases, may cause an idiosyncratic reaction (DILD), usually with a mixed cholestatic/hepatocellular profile. He further concluded that although there may be some underreporting of severe liver reactions worldwide, it is very unlikely that there should be a significant underreporting in Western Europe. The few reported severe side effects, despite the widespread use of ashwagandha, indicate that such reactions must be very rare, probably much rarer than the equally rare, lethal or transplant-requiring idiosyncratic reactions to commonly used drugs (such as ibuprofen, diclofenac and paracetamol), which can be purchased over the counter.

Although ashwagandha root, at first glance, seems to be very safe, one must be watchful of the few case reports indicating ashwagandha root toxicity, especially when used in combination with other herbal remedies. In some of these reports, the root extract combined with extracts from the leaves may be responsible for liver toxicity [106,107,108,109,110]. Thus, adulterations of the product, as well as the administration of high doses, have been suggested as possible explanations for the toxicity reported in some of the cases [111]. However, in rare instances where ashwagandha root extract was taken as a monotherapy, liver injury has been reported, and possible biochemical pathways for the rare phenomenon have been discussed [107,108,109,110]. It should, however, be mentioned that for all the cases reported above, except for one, the liver injury was self-limiting [110]. A summary of different case reports on liver injury that could have been induced by ashwagandha treatment includes symptoms like jaundice and pruritus and elevated levels of key liver enzymes and markers (ALT, AST and bilirubin), which can occur after a few weeks of treatment but also after daily administrations lasting up to several months. As indicated earlier, values for the liver function test reverted to normal levels within 1 to 8 months of cessation of ashwagandha treatment in all the reported cases reviewed in this study, apart from one case. Nevertheless, there are no clear indications as to what could be the metabolic mechanisms or nature of the substances that cause disturbances of the liver in the very few reports of side effects associated with ashwagandha treatment [107,108,109,110]. While discussing possible threats from ashwagandha treatment and how we should respond to them, Simon Mills [111] points out that idiosyncratic reactions known from consuming general food, herbs or medicine could play a role in some of these rare cases. Underlying enzyme deficiencies from genetic or hormonal variations have been known to cause reactions, and some can form a DNA adduct from unknown sources. In addition, low levels of glutathione in the individual, as mentioned earlier, may contribute to the “idiosyncratic reactions”. As the liver is the main site for drug metabolism, many drugs are bio-transformed there, and some of the toxic phytochemicals may harm the liver cells if the glutathione level is low. The combination of drugs (paracetamol) and alcohol can also cause severe liver injury. To the best of my knowledge, we do not know the pattern of possible (if any) interactions between ashwagandha and over-the-counter and prescription medicines, especially in patients who are prone to marginal or more severe liver injury. It might also make life easier (and safer) if it were mandatory to declare the content of withaferin A and withanone (mainly present in the leaves) on each package entering the shelves in a store.

The sales of KSM66 are approximately 2.5 billion doses per year, of which 650,000 doses are consumed in Europe. In the USA, the world’s largest market of ashwagandha outside India, KSM66 accounts for 49% of the total sales (spin data).

From these figures, one would expect a far higher number of reported liver injuries than what has been described if the intake of ashwagandha was truly associated with serious liver side effects and should be avoided [106,107,108,109,110,111,112,113]. Interestingly, some of the reported side effects mentioned above were from patients who already had severe liver injury prior to taking ashwagandha. These findings should also be seen in light of daily reported liver damage from high intake of alcohol or from paracetamol [114] and NSAIDs [115,116], which can be bought without a prescription in most parts of the world. It is also important to note that in animal models, ashwagandha has never been observed to confer liver toxicity. When animals were treated with 5 to more than 200 times the dose of what is relevant for humans, there were no histopathological changes in the different tissues and organs tested and no signs of deleterious or disruptive changes in blood biochemistry [94,97,117,118]. By contrast, there was an improvement in the level of antioxidants like superoxide dismutase and catalase in hepatic tissue [72,73,75], indicating some liver protection with ashwagandha treatment, including protection from paracetamol-induced liver damage [72] and non-alcoholic steatohepatitis [76,77], possibly by improving glutathione levels. As glutathione was shown to protect the liver in humans suffering from alcohol-induced liver injury [82], improvement of glutathione levels may be important. Therefore, the thorough evaluation of this issue and deep diving into the literature regarding side effects and ashwagandha treatment are blurred and lack any cohesive conclusion. Ashwagandha seems to exert some liver protection, and at the same time, there are rare case reports in a few individuals where the changes in liver enzymes tend towards liver injury. Still, other more likely reasons for liver injury could be multiple, like adulteration of the product (possibly contamination with ashwagandha leaves or administration of very high doses, as well as combination with other herbs or medicines, which are dangerous to compromised livers). Finally, there is the possibility of idiosyncratic reactions in sensitive or predisposed individuals. Therefore, there is a need for more studies to elucidate both the safety and efficacy of ashwagandha products.

#### Summary of Declared Case Reports of Ashwagandha Treatment with Side Effects

**India:** Eight cases were reported [106]. Three of these cases were known to have pre-existing chronic liver disease, while two cases were diagnosed to have underlying chronic liver failure during the evaluation of herb-induced liver injury (HILI). Among the latter two, one was diagnosed with HILI on the background of non-alcoholic steatohepatitis (NASH) on liver biopsy, while the other had imaging features of cirrhosis and portal hypertension. One patient had a history of non-Hodgkin’s lymphoma in remission. The various types of ashwagandha formulations consumed by patients included homemade powdered roots, jam preparations, manufactured branded ashwagandha root formulations as powdered root and syrups and nonbranded Ayurveda practitioner-prepared tablets.

Dosing in the different cases was not clear. Time to onset of symptoms and lab abnormalities varied from 14 to 540 days. The major symptom was jaundice, and biochemical disturbances were elevated liver enzymes and elevated bilirubin. Four had liver injury on a cholestatic basis, three on a hepatocellular basis, and one was mixed. Of the four patients with chronic pre-existing liver disease, three died with the development of acute-on-chronic liver failure (ACLF), while the one without ACLF survived. The mortality rate in ACLF is up to 89%. In all the patients, ashwagandha liver injury was self-limited, except in the ones who died from their pre-existing or underlying chronic liver failure, and where it was unlikely that ashwagandha was the cause of death [106].

**USA:** Six cases have been reported up to 2021, of which five were reversible [107] and one ended up with liver transplantation [109]. The one patient with liver transplantation was treated years earlier with thyroid hormones due to thyroid cancer. She went to an herbal practitioner three months before being admitted to the hospital, as she felt tired and was not in good shape. She was treated with ashwagandha (unknown dose) at the same time as she was taking corticoids. After two months, she discontinued treatment with ashwagandha as she felt the treatment was not working. One month into the ashwagandha treatment, she had gone to the hospital because, in her opinion, things were not getting better. It turned out that she had elevated liver enzymes and bilirubin, which continued to increase when measured a month later. The situation slowly got worse and ended with her getting a liver transplant. It is not easy to understand why liver enzymes and bilirubin were elevated one month after withdrawal of ashwagandha—instead, the enzymes continued to increase during the ashwagandha withdrawal period. Her case was different from other reviewed cases, where normalization of liver enzymes and bilirubin followed withdrawal of Ashwagandha treatment [109].

**Iceland:** Five cases were reported in 2020; of these, three were from Iceland [110]. After withdrawal of ashwagandha, the previously high levels of liver enzymes and bilirubin were normalized. The formulations of the ashwagandha used, as well as the presence or absence of multiple medications, are not known. In addition, contaminants from ashwagandha leaves could not be excluded in some of the cases [106,111].

**Ireland:** One case reported [107]. Indication: anxiety. A 39-year-old woman, 3 months abstinent from alcohol, bought over-the-counter ashwagandha together with basil and biotin. She developed cholestatic hepatitis after 45 days of treatment. However, she had remission one month after withdrawal of ashwagandha treatment.

**Holland:** From 2018 to 2023, four cases were reported [112]. Indications are stress, cognitive disorder and panic reaction. Symptoms started after 3–10 months of treatment. Although all four cases were being treated with other herbal remedies/vitamins and medicine, it was ashwagandha that was suspected to have caused the liver injury. The blood biochemistry of the patients indicated jaundice and elevated liver enzymes. Three recovered after 1 month of withdrawal of ashwagandha, but in one patient, remission occurred after three months following withdrawal.

Since 2023, the Netherlands Pharmacovigilance Centre, Lareb, reported an additional eight cases of liver toxicity associated with the consumption of products that contained ashwagandha. The interpretation of these reports is not so easy. In some of the cases, there is the possibility that ashwagandha administration to people with certain underlying liver abnormalities could lead to the elevation of liver enzymes [119,120].

**Japan:** One case, a 20-year-old man, who took more than double the recommended dose for one month, was admitted to the hospital with cholestasis. One hundred and fifty days after withdrawal of treatment, he totally recovered [107].

**Poland:** One case, a 23-year-old man, who had taken ashwagandha as a single-ingredient, over-the-counter supplement for 3 months (dose not reported). Complete resolution within 106 days of ashwagandha withdrawal [106,107].

**Germany:** One case, a 65-year-old female taking ashwagandha as a single ingredient for 30 days, though the dosage is not reported. Complete resolution within 2 months after withdrawal [107].

Underlying ailments include prior daily exposure to alcohol, concomitant with intake of over-the-counter or prescription medicine, as well as simultaneous self-medication with different herbs. These aspects, together with the fact that the dosage of ashwagandha and the types of other medication were not stated, make it difficult to draw a clear causal relation to ashwagandha treatment.

Unpredictable liver injuries (hepatoxicity) caused by defined chemicals (medicine) are well known, and herb-induced liver injury, although not common, can be caused by one or more of the phytochemicals and, in rare cases, contribute to unpredictable hepatoxicity. A toxic effect of a drug, herbal remedy or food would normally be predictable; have a shorter and well-defined latency period; and should be reliably reproduced in animal models. This is not the case for ashwagandha, where reports of side effects are so far unpredictable, the latency period varies from 14 to 540 days, and animal models have not indicated liver toxicity. Idiosyncratic injury is difficult to predict, varies in latency and can be linked to immune responses in a susceptible patient [107,108].

One possible explanation of reported liver injury from ashwagandha can be that a herb-specific compound, for example, withanone, which is present, to some extent, in the root and, to a much larger degree, in the leaves (see Table 1 and Table 2), can form DNA adducts and interfere with biological activity in a few susceptible individuals, especially when glutathione is low. The process may be reversible and interfere with amines [79,80,81,82,83]. At the same time, one must admit that there is clear evidence that ashwagandha can protect the liver (i.e., lower the liver enzymes AST and ALT in the background of paracetamol abuse), as well as protect the liver against steatosis [77] and anti-peroxidation of hepatic tissue [74] and protect from histopathological changes [75].

When a dose of 600 mg of ashwagandha (KSM66) daily was tested in a placebo-controlled study lasting for two months, there were no changes in the liver enzymes ALT or AST when comparing the initial levels to the two-month levels, nor when comparing the placebo and active treatment [29]. Recently, a 12-month placebo-controlled study on the safety of ashwagandha (KSM66) was released, indicating no significant clinical changes in liver parameters such as AST and ALT [30].

A three-month study on Wistar rats administered doses of ashwagandha (KSM66) up to 2000 mg/kg of body weight/day [94] and indicated no alteration in AST, ALT or bilirubin as a result of treatment. The findings in this study were further confirmed by histopathological examination of the liver, which did not reveal any lesions when the treated animals were compared to control animals [94].

In conclusion, there have been a few reports worldwide in which ashwagandha has been claimed to have caused liver injury. Interestingly, this is irrespective of who the producer is or where the product originates. In the few cases reported, the dosage used has sometimes been unknown, and where it was stated, it has often been above the recommended daily dose of 600 mg. However, in a few cases where ashwagandha was monotherapy and the dose was <600 mg daily, jaundice, pruritus and elevated laboratory values for AST, ALT and bilirubin have been reported. One to eight months after withdrawal of ashwagandha treatment, symptoms and laboratory values were normalized, except in one person with comorbidities. As stated earlier in this paper, millions of doses of ashwagandha are consumed every day worldwide. Indeed, the exact number of daily doses of ashwagandha sold outside India is hard to estimate. According to Ixoreal Biomed, the producer of KSM66, 2.5 billion daily doses of KSM66 are sold outside India every year. Of this, 650 million daily doses are sold in Europe. In the background of this high global exposure, the reported side effects of ashwagandha are rare, indicating the general safety of a well-defined root extract with a negligible content of withaferin A and withanone. The safety of the high-quality root extract of ashwagandha is also reinforced by the fact that in a total of 40 clinical studies with KSM66, the number of reported side effects was comparable (4%) in both the placebo and active groups. Furthermore, most of the side effects were mild and transient [119,120,121].

### 3.10. Effect Size

There is clear evidence from the reviewed literature, 40 placebo controlled clinical trials, that ashwagandha positively impacts mood, sleep, the cardiovascular system, the immune system and endocrine functions. However, the effect size (ES) has not been estimated in any of the studies. When looking broadly at the data, the ES of ashwagandha on anxiety and sleep [35] is not far from what can be expected when anxiety and sleep patients are treated with benzodiazepines [122]. As benzodiazepines are often associated with the development of addiction and other undesirable side effects, it would be of great interest to investigate and compare the ES of ashwagandha to other prescription medicines used for the areas where ashwagandha treatment shows promise. If herbal remedies (green therapy) can reduce the consumption of prescription medicine, including those like benzodiazepines, this would be of great interest to our society in general and to governmental organizations that determine which health alternatives should be promoted.

## 4. Limitations of This Study

As a narrative review, a major limitation of this study is the heterogeneity in the reviewed literature. Although a structured literature search was carried out for publications in Google Scholar using the search word “ashwagandha”, the material collected included publications spanning nearly three decades (1998 to the present). The literature considered included published basic scientific studies of ashwagandha effects in cells and different animal models, as well as in human intervention studies and clinical trials with varying designs, protocols and outcome measures. Consequently, this study only provides a contextual basis for more systematic studies.

## 5. Conclusions

All of the literature accessed for this review indicates that ashwagandha root powder, or extracts thereof, can improve the immune system, sex hormones and libido and the uptake of oxygen, as well as improve muscle strength. Ashwagandha can also positively impact stress, anxiety, insomnia and sleep and cognitive function. There are indications that ashwagandha can boost thyroid function in modest hypothyroid volunteers, while up until now, there are no indications that ashwagandha will improve thyroid hormones above normal levels in volunteers with normal thyroid function who are treated for up to twelve months. However, more long-term studies are still welcome to shed more light on this question. Ashwagandha is also reported to improve the reproductive system, especially in men, and to protect the liver.

In general, it should be noted that side effects with ashwagandha are rare. Indeed, the levels reported so far in clinical trials are comparable to what would be seen with a placebo. A few cases indicating liver injury have been reported during ashwagandha treatment, even when the dose is <600 mg daily. Symptoms like jaundice, pruritus, elevated liver enzymes and the elevation of bilirubin normalized after withdrawal of treatment in all the cases except one, who had been treated earlier for a cancer and had received glucocorticoids. Conversely, ashwagandha is also reported to protect the liver, making it difficult to decipher the basis for liver injury reported in the rare cases mentioned above. However, idiosyncratic reactions that have also been reported for other herbs and medicines may be at play here. Other likely explanations include the possibility that a few volunteers who present with the rare side effects of liver injury may have enzyme deficiencies from genetic or hormonal variation, as exemplified in volunteers low in glutathione. In the few cases where hyperthyroid function was reported, symptoms and hormone levels normalized shortly after withdrawal of ashwagandha treatment.

Overall, administrations of ashwagandha root powder and extracts seem to be safe. Due to the lack of product control and the risk of adulteration (for example, the inclusion of the less expensive leaves in the preparations), it is wise to only use well-standardized and clinically documented ashwagandha root products. Moreover, there is a need for more randomized, double-blinded, placebo-controlled human clinical trials that clarify the efficacy, safety and effect size of standardized ashwagandha root preparations. Animal model studies aimed at clarifying the molecular basis of its action will also be very useful. A systematic biological approach is highly recommended for studying the herbal remedy ashwagandha, especially because of its broad spectrum of different activities.

## Figures and Tables

**Table 1:** The amounts of different constituents in roots, leaves and fruits.

Metabolite	Roots	Leaves	Roots and Leaves	Fruits	Reference
**Primary metabolites**					
**Fatty acids**					
Palmitic acid	1.18 ± 0.2	3.55 ± 0.5			[9]
Oleic acid	0.39 ± 0.1	0.71 ± 0.1			[9]
Linoleic acid	1.31 ± 0.2	1.52 ± 0.2			[9]
Linolenic acid	0.15 ± 0.1	4.38 ± 0.5			[9]
**Amino acids**					
Alanine		Detected			[9]
Aspartate		Detected			[9]
Asparagine			Detected		[9]
Glutamine			Detected		[9]
Isoleucine		19.83 ± 0.8			[9]
Lysine		Detected			[9]
Leucine		Detected			[9]
Phenylalanine		Detected			[9]
Tyrosine		Detected			[11]
Threonine		Detected			[11]
Ornithine		21.5 ± 0.8			[9]
Valine		5.60 ± 0.5			[11]
**Monosaccharide**					
Galactose			Detected		[9]
α-Glucose		6.11 ± 0.5			[9]
Β-Glucose		10.22 ± 0.9			[9]
**Alcohol**					
Glycerol	Detected				[9]
**Organic acid**					
Lactic acid		Detected			[9]
Tartaric acid		4.10 ± 0.4			[11]
**Other compounds**					
Citric acid		Detected			[9]
Fructose-5	Detected				[9]
Fumaric acid		0.6 ± 0.2			[9]
GABA	Detected	16.74 ± 0.8			[9]
Glutamate			Detected		[9]
Succinate		12.75 ± 0.5			[9]
**Secondary metabolites**					
**Triterpenoids (Steroidal lactones)**					
Withanone	5.54 ± 0.4	18.42 ± 0.8			[9]
27-deoxywhitanone	3.94 ± 0.4	1.63 ± 0.2			[9]
27-hydroxywithanone			0.50 ± 0.1		[9]
Withaferin A	0.92 ± 0.4	22.31 ± 1			[10,11]
17-hydroxy-27-deoxy- Withaferin A	0.66 ± 0.2	3.61 ± 0.5			[9]
Withanolide A	3.88 ± 0.7	2.11 ± 0.5			[9,10]
Withanolide B-D					[12]
27-hydroxy Withanolide B	0.55 ± 0.2	2.78 ± 0.5			[9]
Withanoside IV	0.44 ± 0.1	1.60 ± 0.2			[9]
Withanoside VI	3.74 ± 0.2	1.90 ± 0.2			[9]
12-deoxywithastromonolide	1.90 ± 0.5	2.15 ± 0.5			[9]
Physagulin	Not detected	3.46 ± 0.4			[9]
**Flavonoids**					
Kaempferol	Not detected	Not detected		0.06	[13]
Naringenin	Not detected	Not detected		0.50	[13]
(+)-Catechin	12.82	28.38		19.48	[13]
**Phenols**					
Gallic acid	Not detected	0.18		Not detected	[13]
Syringic acid		0.30			[13]
p-coumaric acid		0.80			[13]
vanillic acid		0.15			[13]
benzoic acid		0.80			[13]
**Alkaloids**					
Trigonelline		1.33 ± 0.3			[9]
Choline			3.53 ± 0.5		[9]
Uracil		3.90 ± 0.2			[11]

There is a huge variation in the different constituents when comparing the leaf and root. The numbers of references are given in the right panel. Units throughout are the mg/g dry weight.

**Table 2:** Total metabolic content in leaves and roots.

Extract Partition	Total Metabolite Content (mg/g of Dry Weight)	
	Leaf	Root
Hexane	34.29 ± 2.0	4.44 ± 0.8
Chloroform	35.71 ± 1.5	10.00 ± 1.0
n-butanol	28.57 ± 1.6	11.11 ± 1.2
Methanolic water	228.57 ± 5.2	15.00 ± 1.6

Studies have shown that for different extracts of *Withania somnifera* L., the quantity of the metabolites in leaves and roots is very different from each other, particularly in the aqueous methanolic fraction [9].

## Data Availability

No new data were created or analyzed in this study.
